# AGO Breast Panel Recommendations for the use of treatment-relevant biomarkers in early and advanced breast cancer—2026 Update

**DOI:** 10.1016/j.breast.2026.104873

**Published:** 2026-07-17

**Authors:** Andreas Hartkopf, Ute-Susann Albert, Rupert Bartsch, Ingo Bauerfeind, Maggie Banys-Paluchowski, Vesna Bjelic-Radisic, Jens-Uwe Blohmer, Peter Dall, Eva Maria Fallenberg, Peter A. Fasching, Michael Friedrich, Oleg Gluz, Nadia Harbeck, Jörg Heil, Juliane Hörner-Rieber, Jens Huober, Wolfgang Janni, Hans-Heinrich Kreipe, David Krug, Thorsten Kühn, Sherko Kümmel, Sibylle Loibl, Diana Lüftner, Michael Patrick Lux, Nicolai Maass, Marion Tina van Mackelenbergh, Christoph Mundhenke, Tjoung-Won Park-Simon, Mattea Reinisch, Toralf Reimer, Kerstin Rhiem, Achim Rody, Andreas Schneeweiss, Florian Schütz, Hans-Peter Sinn, Christine Solbach, Erich-Franz Solomayer, Elmar Stickeler, Marc Thill, Michael Untch, Isabell Witzel, Achim Wöckel, Rachel Wuerstlein, Nina Ditsch, Tanja Fehm, Volkmar Müller, Marcus Schmidt

**Affiliations:** a Department für Frauengesundheit, Universitätsfrauenklinik, Tübingen, Germany; b Klinik für Frauenheilkunde und Geburtshilfe, Universitätsklinikum Würzburg, Würzburg, Germany; c Klinik für Innere Medizin I, Abteilung für klinische Onkologie, Medizinische Universität Wien, Austria; d Frauenklinik und Brustkrebszentrum LA-Regio-Klinik, Landshut-Mitte, Germany; e Klinik für Gynäkologie und Geburtshilfe, Universitätsklinikum Schleswig-Holstein, Campus Lübeck, Lübeck, Germany; f Brustzentrum und Abteilung Senologie, Helios Universitätsklinikum Wuppertal, University Witten/Herdecke, Germany; g Gynäkologie mit Brustzentrum, Charité-Universitätsklinikum Berlin, Berlin, Germany; h Frauenklinik, Städtisches Klinikum Lüneburg, Lüneburg, Germany; i Institut für Klinische Radiologie, Klinikum der Universität München Campus Großhadern, Munich, Germany; j Universitätsfrauenklinik, Universitätsklinikum Erlangen, Erlangen, Germany; k Klinik für Frauenheilkunde und Geburtshilfe, Helios Klinikum Krefeld, Krefeld, Germany; l Brustzentrum Niederrhein, Evang. Krankenhaus Johanniter Bethesda, Mönchengladbach, Germany; m Brustzentrum, Klinik und Poliklink für Frauenheilkunde und Geburtshilfe, University Hospital Munich, LMU und CCC Munich, Munich, Germany; n Brustzentrum Heidelberg, Klinik St. Elisabeth, Heidelberg, Germany; o Klinik für Strahlentherapie und Radioonkologie, Universitätsklinikum Düsseldorf, Düsseldorf, Germany; p Brustzentrum, Kantonspital St. Gallen, St. Gallen, Switzerland; q Klinik für Frauenheilkunde und Geburtshilfe, Universitätsklinikum Ulm, Ulm, Germany; r Institut für Pathologie, Medizinische Hochschule Hannover, Hanover, Germany; s Klinik für Strahlentherapie, Universitätsklinikum Schleswig-Holstein, Campus Kiel, Kiel, Germany; t Filderklinik, Filderstadt, Germany; u Klinik für Senologie, Evangelische Kliniken Essen Mitte, Essen, Germany; v GBG Forschungs GmbH, Neu-Isenburg / Goethe Universität, Frankfurt am Main, Germany; w Fachklinik für Onkologische Rehabilitation, Immanuel Hospital Märkische Schweiz, Buckow und Immanuel Hospital Rüdersdorf / Medical University of Brandenburg Theodor Fontane, Rüdersdorf, Germany; x Klinik für Gynäkologie und Geburtshilfe, St. Vincenz Kliniken Salzkotten & Paderborn, Germany; y Klinik für Gynäkologie und Geburtshilfe, Universitätsklinikum Schleswig-Holstein, Campus Kiel, Kiel, Germany; z Klinik für Gynäkologie und Geburtshilfe, Klinikum Bayreuth, Bayreuth, Germany; aa Klinik für Frauenheilkunde und Geburtshilfe, Medizinische Hochschule Hannover, Hanover, Germany; ab Frauenklinik, Universitätsklinikum Mannheim, Mannheim, Germany; ac Universitätsfrauenklinik und Poliklinik am Klinikum Südstadt, Rostock, Germany; ad Zentrum Familiärer Brust- und Eierstockkrebs, Universitätsklinikum Köln, Cologne, Germany; ae Nationales Centrum für Tumorerkrankungen, Universitätsklinikum und Deutsches Krebsforschungszentrum, Heidelberg, Germany; af Klinik für Gynäkologie und Geburtshilfe, Diakonissen Krankenhaus Speyer, Speyer, Germany; ag Sektion Gynäkopathologie, Pathologisches Institut, Universitätsklinikum Heidelberg, Heidelberg, Germany; ah Brustzentrum, Klinik für Frauenheilkunde und Geburtshilfe, Universitätsklinikum Frankfurt, Frankfurt, Germany; ai Klinik für Frauenheilkunde, Geburtshilfe und Reproduktionsmedizin, Universität des Saarlandes, Homburg, Germany; aj Klinik für Gynäkologie und Geburtsmedizin, Universitätsklinikum Aachen und CIO ABCD, Aachen, Germany; ak Klinik für Gynäkologie und Gynäkologische Onkologie, Agaplesion Markus Krankenhaus, Frankfurt, Germany; al Helios Klinikum Berlin-Buch, Studienkoordination Brustzentrum und gynäkologisch- onkologisches Zentrum, und Medical School Berlin, Berlin, Germany; am Klinik für Gynäkologie, Universitäts Spital, Zürich, Switzerland; an Klinik für Frauenheilkunde und Geburtshilfe, Universitätsklinikum Augsburg, Augsburg, Germany; ao Klinik für Gynäkologie und Geburtshilfe, Universitätsklinikum Düsseldorf, CIO ABCD, Düsseldorf, Germany; ap Klinik und Poliklinik für Gynäkologie, Universitätsklinikum Hamburg-Eppendorf, Hamburg, Germany; aq Klinik und Poliklinik für Geburtshilfe und Frauengesundheit der Johannes-Gutenberg-Universität Mainz, Mainz, Germany

**Keywords:** Breast cancer, Systemic treatment, Biomarker, Translational research, AGO recommendations

## Abstract

Biomarkers are integral to modern breast cancer management by providing essential information for prognosis and treatment selection. This review presents the 2026 update of the recommendations of the Arbeitsgemeinschaft Gynäkologische Onkologie (German Gynecological Oncology Group, AGO) on therapy-relevant prognostic and predictive biomarkers. The recommendations are based on a structured evaluation of the most recent and clinically relevant evidence using the AGO grading system to support decision-making in routine clinical practice.

## Highlights

• Biomarkers are increasingly used to guide therapeutic decision-making.
• The AGO Breast Expert Panel provides annual recommendations using a 5-level rating system.
• This review summarizes all treatment-relevant recommendations on prognostic and predictive biomarkers in early and metastatic breast cancer.

## Graphical abstract

**Figure:**
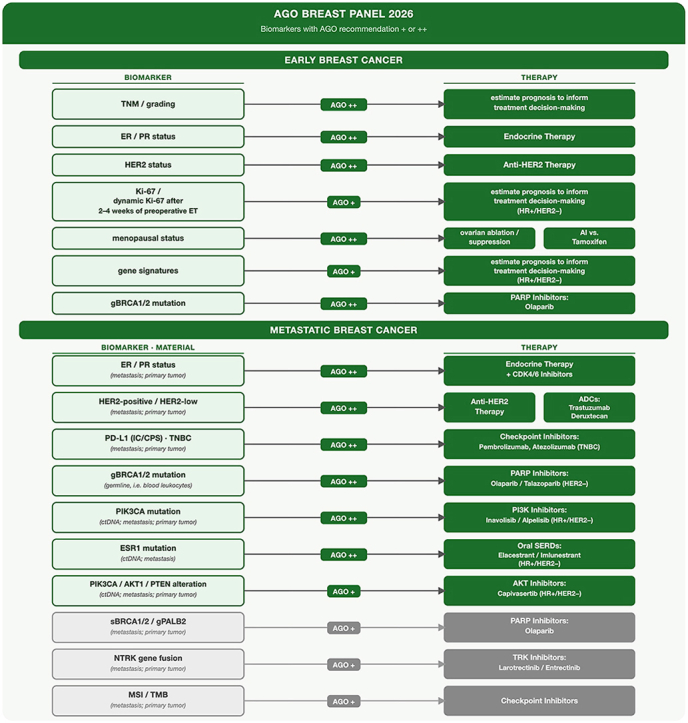

## 1 Introduction

In modern breast cancer (BC) treatment, biomarkers are increasingly used to guide therapeutic decision-making. Prognostic biomarkers provide information on the risk of recurrence, disease progression, or death and are independent of the administered treatment. Nevertheless, prognostic information is therapeutically relevant, as it is essential to estimate an absolute treatment benefit, thereby assessing the need for a (potentially toxic) oncologic therapy (“Who requires therapy?”). In contrast, predictive markers forecast the likelihood of response to a given treatment and are therefore used to also determine relative therapy efficacy (“Does a therapy work?”). Many biomarkers have both prognostic and predictive value.

The following overview summarizes the recommendations of the Breast Expert Panel of the Arbeitsgemeinschaft Gynäkologische Onkologie (AGO; German Gynecological Oncology Group) regarding therapy-relevant prognostic and predictive biomarkers, which are updated annually. This update is based on a structured evaluation algorithm. The most recent and clinically relevant publications are systematically reviewed, and the strength of each recommendation is defined using the 5-level AGO rating system, which provides recommendations for use in clinical practice (Table 1). The focus is on clinically relevant recommendations and those associated with approved therapeutic consequences.

**Table 1:** AGO recommendation levels.

++	This investigation or therapeutic intervention is highly beneficial for patients, can be recommended without restrictions, and should be performed
+	This investigation or therapeutic intervention has limited benefit for patients and can be performed
+/−	This investigation or therapeutic intervention has not shown benefit for patients and may be performed only in individual cases. According to current knowledge, a general recommendation cannot be given
−	This investigation or therapeutic intervention can be of disadvantage to patients and might not be performed
−−	This investigation or therapeutic intervention is of clear disadvantage to patients and should be avoided or omitted in any case

## 2 Patient characteristics, tumor stage, and histopathologic factors

Age (AGO ++), menopausal status (AGO ++), body mass index (BMI, AGO +), concomitant medications, physical and psychological comorbidities, performance status, as well as the patient's preferences are key factors in determining an optimal treatment strategy [1,2].

In early BC (eBC), local tumor extent in the breast (T stage, AGO ++), involvement of ipsilateral lymph nodes (N category, AGO ++), and resectability (R category, AGO +) determine the risk of disease recurrence and distant metastasis [3,4]. Moreover, (y)TNM staging after neoadjuvant systemic therapy (NAT) is used to assess treatment response and thereby allows further refinement of prognosis. In particular, pathological complete response (pCR) in the breast and lymph nodes (AGO ++), as well as the CPS-EG score (AGO +), are used to guide postneoadjuvant systemic treatment [[5], [6], [7]]. Table 2 summarizes treatment relevant biomarkers and their clinical implications in patients with eBC.

**Table 2:** Treatment-relevant biomarker in early breast cancer.

Population	Biomarker	Consequence
All patients	Menopausal status (AGO ++)	Type of Endocrine treatment
All patients	Disease staging and pathological assessment (AGO ++)	Use and intensity of chemotherapy
		Intensity of endocrine treatment
		Use and intensity of radiotherapy
		Use of Ribociclib^a^/Abemaclcib^b^
		Use and and intensity of HER2-directed treatment^c^
		Use of Pembrolizumab^d^
		Use of Olaparib^e^
All patients	ER, PR, HER2, (dynamic) Ki67 (AGO ++)	Endocrine treatment (if HR+)^f^
		Chemotherapy (if high risk)
		Ribociclib (if intermediate-high risk HR+/HER2-)^a^
		Abemaciclib (if high risk HR+/HER2-)^b^
		HER2-directed treatment (if HER2+)^c^
		Pembrolizumab (if high risk TNBC) d
HR+/HER2- AND N0-1 AND therapeutic relevance	Gene expression arrays (AGO +)	Chemotherapy (if high risk) g
		Ribociclib (if high risk)^a^
family history OR therapeutic relevance	gBRCA1, gBRCA2 (AGO ++)	Olaparib (if high risk) e

a Three years of adjuvant ribociclib is indicated in patients with ≥1 positive lymph node or in node-negative patients with T2 disease and high-risk tumor biology (Ki-67 > 20%, grade 3, or a high-risk gene expression signature), or with T3 disease.

b Two years of adjuvant abemaciclib is indicated in patients with ≥4 positive lymph nodes or in patients with 1–3 positive lymph nodes and either grade 3 disease or a tumor size ≥5 cm.

c In stage II–III HER2-positive disease, NAT combined with dual HER2 blockade (trastuzumab and pertuzumab) is the preferred treatment option. Patients achieving pCR should continue trastuzumab for a total duration of 1 year, combined with pertuzumab in case of initial node-positive disease. T-DM1 is indicated for patients who do not achieve pCR. Neratinib may be considered in patients with HR+/HER2+ disease.

d Pembrolizumab is indicated in patients with stage II–III TNBC in combination with neoadjuvant chemotherapy (taxane plus carboplatin followed by AC/EC), irrespective of PD-L1 status, and should be continued after surgery for an additional 9 cycles.

e Olaparib is indicated in patients with germline BRCA1 or BRCA2 mutations and triple-negative disease with residual invasive disease after neoadjuvant chemotherapy or stage II–III disease treated with adjuvant chemotherapy. In HR+/HER2-disease, olaparib is indicated in patients with a CPS-EG score ≥3 after neoadjuvant chemotherapy or ≥4 positive lymph nodes if treated with adjuvant chemotherapy.

f The benefit of endocrine therapy in tumors with low ER expression (1–10%) is uncertain.

g Gene expression assays should be interpreted in conjunction with established clinicopathological risk factors.

Histopathologic prognostic factors include histology (AGO ++), tumor grade according to Elston and Ellis (AGO ++), as well as multiple immunohistochemical markers, including estrogen receptor (ER), progesterone receptor (PR), and human epidermal growth factor receptor 2 (HER2), which are discussed in the next chapter [1,8,9]. Various online prognostic tools (e.g., PREDICT, CTS-5) integrate multiple prognostic factors to estimate recurrence risk (AGO +) [10,11].

The presence of distant metastases (M stage) distinguishes a curative from a non-curative treatment setting, which is associated with fundamentally different therapeutic objectives (AGO ++). In metastatic BC (mBC), overall survival is determined by tumor burden, the pattern of metastatic involvement (e.g. cerebral, visceral, non-visceral, bone-only), and the time point of metastatic occurrence (de novo versus secondary metastatic disease) [12]. Moreover, disease-free interval as well as the number and duration of response to prior treatment lines are important for guiding therapeutic decisions and estimating overall survival. For ER/PR-positive BC, endocrine-sensitive and endocrine-resistant disease are defined according to the timing of relapse and disease progression. Primary endocrine resistance is defined as relapse within the first 2 years of adjuvant endocrine treatment or disease progression within the first 6 months of first-line endocrine treatment for mBC. Secondary (acquired) endocrine resistance is defined as relapse while on adjuvant endocrine treatment after the first 2 years, relapse within 12 months after completion of adjuvant endocrine treatment, or disease progression after 6 months of endocrine treatment for mBC [12].

## 3 Immunohistochemical markers

In clinical practice, BC subtyping is based on the assessment of hormone receptors (HR), namely the estrogen receptor (ER) and progesterone receptor (PR), as well as the human epidermal growth factor receptor 2 (HER2) status [13]. This classification defines the subtypes HR-positive/HER2-negative (often referred to as luminal-like BC), HER2-positive/HR-positive as well as HER2-positive/ER-negative, and triple-negative BC (TNBC), which determine prognosis and treatment algorithms. Of note, intrinsic subtypes (i.e., luminal A, luminal B, HER2-enriched, and basal-like) may overlap with these histopathologic subtypes but are defined on a molecular basis (gene expression patterns, e.g., the PAM50 assay) and can therefore only be approximated using classical immunohistochemistochemmical staining [1]. As HR and HER2 status may change during the course of disease, histological reassessment of recurrent or metastatic lesions should be performed whenever feasible (AGO ++) [14,15].

The ER/PR status is of prognostic relevance (AGO ++) [16,17], predictive of response to endocrine therapy (AGO ++) [18,19] and, when negative or low positive, is associated with better response to NAT (AGO ++) [20]. Classification of a carcinoma as ER- or PR-positive requires at least 1% positive tumor cell nuclei. As there is limited data on benefit from endocrine therapy in tumors with 1–10% positive cells, these tumors should be reported as “ER Low Positive” and considered separately in treatment planning (AGO ++). Interpretation of immunohistochemical staining results should follow the guidelines of the American Society of Clinical Oncology (ASCO) and the College of American Pathologists (CAP) [21].

HER2 testing should also follow the recommendations of the ASCO/CAP guidelines [22]. The HER2 status is considered positive when immunohistochemical staining yields a score of 3+, defined by intense, complete circumferential membrane staining in more than 10% of invasive tumor cells. In cases with a score of 2+ (weak to moderate complete membrane staining in >10% of invasive tumor cells), additional in situ hybridization (CISH, SISH, or FISH) is required (AGO ++) [22]. Tumors with a score of 3+ or 2+ with positive in situ hybridization are classified as HER2-positive. HER2-positive tumors respond to HER2-targeted therapies, including trastuzumab, pertuzumab, tucatinib, neratinib, lapatinib, and HER2-directed antibody–drug conjugates (ADCs), such as trastuzumab emtansine (T-DM1) and trastuzumab deruxtecan (AGO ++). HER2 status is also associated with prognosis (AGO ++) and response to NAT (AGO ++) [23]. In metastatic breast cancer (MBC), the ADC trastuzumab deruxtecan is effective also in tumors with low (1+ or HER2 2+ with ISH-negative) and ultralow (HER2 0 with faint membrane staining) HER2 expression (AGO ++) [[24], [25], [26]].

Ki-67 is a proliferation marker that correlates with pCR rates following NAT (AGO +) [27,28]. Due to its prognostic properties, it is used as a decision aid for or against (neo)adjuvant chemotherapy in HR-positive/HER2-negative EBC. Endocrine response can be assessed by analyzing changes in Ki-67 expression after a short course (2–4 weeks) of preoperative endocrine induction treatment (AGO +) [29]. A decrease to <10%, in conjunction with other prognostic factors like gen expression markers supports decision-making regarding (neo)adjuvant chemotherapy [30].

The programmed death ligand 1 (PD-L1) is an immune checkpoint molecule that binds to its receptor PD-1, thereby suppressing T-cell activity [31]. PD-L1 is expressed by tumor cells as well as by lymphocytes within the tumor microenvironment. Immune checkpoint inhibitors block the PD-1/PD-L1 interaction, thereby restoring antitumor immune activity. PD-L1 assessment in metastatic TNBC is predictive of response to the checkpoint inhibitors pembrolizumab and atezolizumab [32,33]. Importantly, assay methods and cutoffs are indication- and drug-specific. Pembrolizumab is approved in combination with chemotherapy for metastatic breast cancer patients with a combined positive score (CPS) ≥10 (AGO ++), whereas atezolizumab is used in tumors with an immune cell score (IC score) ≥1. Importantly, the 22C3 combined positive score used for pembrolizumab and the SP142 immune-cell score used for atezolizumab are not interchangeable, as they identify only partially overlapping patient populations [34]. Substitution of one assay for the other is not validated and may therefore result in inappropriate patient selection. For NAT of TNBC, pembrolizumab is approved regardless of PD-L1 expression [35].

## 4 Gene expression signatures

Several prognostic gene expression signatures are commercially available (Table 3). In Germany, Oncotype DX (AGO +), EndoPredict (AGO +), Prosigna (AGO +), and MammaPrint (AGO +) are commonly used in clinical practice. All these genomic assays integrate the activity of multiple genes into a prognostic score and are applied in HR-positive/HER2-negative EBC to support chemotherapy decision making. While Oncotype DX, MammaPrint, and, most recently, Prosigna have been validated for treatment decision-making in prospective randomized controlled phase III trials (TAILORx/RxPONDER, MINDACT, and OPTIMA, respectively), evidence supporting EndoPredict is derived primarily from retrospective analyses of prospective cohorts [[36], [37], [38], [39], [40], [41], [42]]. Patients with invasive EBC of at least grade 2 and at least 20 mm tumor size may additionally be treated with the CDK4/6 inhibitor ribociclib in case of a high-risk genomic assay [43]. Gene expression signatures should always be assessed on untreated tumor tissue (i.e., on core biopsies in the case of endocrine induction therapy) and should be interpreted in the context of established clinical-pathological criteria (menopausal status, tumor size, lymph node involvement, grade, Ki-67, ER, PR, HER2).

**Table 3:** Gene expression signatures.

Gene expression profiles (GEP, multigene assays, gene signatures)	AGO
MammaPrint® (N0-1)	+^a^
Oncotype DX® (N0-1, HR + HER2-)	+^a^
EndoPredict® (N0-1, HR+, HER2 -)	+^a^
Prosigna® (N0-1, HR+, HER2 -)	+^a^
Breast Cancer Index^SM^ (N0-1, HR + HER2-)^b^	+/−b

a Should only be used in the context of clinical-pathological criteria (tumor size, nodal involvement, grade, Ki-67, ER, PR, HER2).

b Estimation of late recurrence.

## 5 Treatment-relevant germline alterations

Therapy-relevant germline alterations in cancer predisposition genes are inherited in an autosomal dominant manner. As these mutations are present in all somatic cells, nucleated peripheral blood cells are used for detection. Pathogenic germline mutations in BRCA1, BRCA2, and PALB2 (gBRCA1, gBRCA2, and gPALB2 mutations) not only increase the risk of developing BC (and other malignancies) but are also predictive of response to systemic therapies, particularly PARP inhibitors (Table 4). The presence of a gBRCA1 or gBRCA2 mutation also increases the likelihood of achieving pCR after neoadjuvant chemotherapy, irrespective of whether carboplatin is included (AGO +) [44]. Therefore, testing for gBRCA1/2 (AGO ++) and gPALB2 (AGO +) alterations is indicated whenever the results may inform systemic treatment decisions, regardless of the familial cancer risk.

**Table 4:** Clinically relevant germline alterations in early and metastatic breast cancer.

Subtype	Marker	Timepoint of assessment^a^	Therapeutic consequence
**Early breast caner**			
HR+/HER2-	gBRCA1 (AGO++) gBRCA2 (AGO++)	after neoadjuvant chemotherapy with non-pCR and CPSEG≥3 or adjuvant chemotherapy and at least pN2	Olaparib, 1year adjuvant in combination with endocrine therapy (AGO++)
TNBC	gBRCA1 (AGO++) gBRCA2 (AGO++)	after neoadjuvant chemotherapy with non-pCR or adjuvant chemotherapy and at least pN1 or pT2	Olaparib, 1year adjuvant (AGO++)
**Metastatic/advanced breast cancer**			
HR+/HER2-	gBRCA1 (AGO++) gBRCA2 (AGO++)	after 1st-Line ET-based treatment	Olaparib or Talazoparib until disease progression (AGO++)
	gPALB2 (AGO++)	simultaneously to BRCA testing	Olaparib until disease progression (AGO+)^b^
TNBC	gBRCA1 (AGO++) gBRCA2 (AGO++)	from 1st line treatment on	Olaparib or Talazoparib until disease progression (AGO++)
	gPALB2 (AGO++)	simultaneously to BRCA testing	Olaparib until disease progression (AGO+)^b^

AGO, Arbeitsgemeinschaft Gynäkologische Onkologie; BRCA1/2, breast cancer1/2 gene; germline (g), HER2, human epidermal growth factor receptor 2; HR, hormone receptor; PALB2, partner and localizer of BRCA2; TNBC, triple negative breast cancer.

a Patients who meet the criteria for an increased familial risk are tested at any time point, regardless of tumor biology or treatment setting.

b not approved.

In patients with a gBRCA1 or gBRCA2 mutation, one year of (postneo-)adjuvant olaparib is indicated in HER2-negative EBC with an increased risk of recurrence (AGO ++) [45]. Risk stratification follows the inclusion criteria of the OlympiA trial: In HR-positive/HER2-negative disease after neoadjuvant chemotherapy, absence of pCR and a CPS-EG score ≥3, or after adjuvant chemotherapy with at least four pathologically involved lymph nodes (≥pN2); in TNBC after NAT in the absence of pCR, or after adjuvant chemotherapy with lymph node involvement (≥pN2) or a tumor size ≥2 cm (≥pT2).

In MBC, gBRCA1/2 or gPALB2 mutations are detected in approximately 6% of patients, independent of family history [46]. In HER2-negative disease, poly (ADP-ribose) polymerase (PARP) inhibitors such as olaparib (OlympiAD trial) and talazoparib (EMBRACA trial) are recommended (HR-: AGO ++/HR + AGO +) [47,48]. Although PARP inhibitors are not approved for gPALB2 mutations, their use is supported by compelling data from a single-arm phase II study (AGO +) [49]. There is also no formal approval for PARP inhibitors in the presence of somatic BRCA1 or BRCA2 mutations (sBRCA1, sBRCA2); however, treatment may be considered on an individual basis, for example after a recommendation of a molecular tumor board (AGO ± for olaparib) [49].

In Germany, any licensed physician may initiate germline genetic testing after obtaining written informed consent in accordance with the Genetic Diagnostics Act (GenDG), provided that the test result is required as a predictive factor for treatment determination. In these cases, additional certification in genetic counseling is not required. If a pathogenic mutation is identified, the patient should subsequently receive formal genetic counseling by a physician with appropriate training.

## 6 Somatic alterations

Somatic genetic alterations arise during tumorigenesis or tumor progression and are therefore restricted to tumor cells; some may occur early in the course of disease, whereas others emerge at later stages or are induced by treatment pressure. Many treatment-relevant alterations affect signal transduction pathways regulating proliferation and differentiation and are particularly relevant for the management of endocrine-resistant HR-positive/HER2-negative MBC [50,51].

The PI3K (phosphatidylinositol 3-kinase)/AKT/PTEN (phosphatase and tensin homolog) signaling pathway regulates cell growth, survival, and metabolic processes. The PIK3CA (phosphatidylinositol-4,5-bisphosphate 3-kinase catalytic subunit alpha) gene encodes a catalytic subunit of PI3K, a key component of this pathway. Therapy-relevant PIK3CA mutations are present in approximately 35–40% of HR-positive/HER2-negative tumors [52]. These are frequently hotspot mutations (especially E542K, E545K, H1047R and H1047L) that remain stable throughout disease progression [53]. Given the high concordance across disease stages, primary tumor tissue, metastatic tissue, or liquid biopsy (ctDNA analysis) may be used for testing. However, as mutations can sometimes also be acquired, current tumor tissue or ctDNA should be preferred whenever possible.

The following PI3K inhibitors are approved for use in HR-positive/HER2-negative MBC with PIK3CA mutations:

–Inavolisib in combination with palbociclib and fulvestrant as first-line therapy in patients with endocrine resistance (recurrence during or within 12 months after adjuvant endocrine therapy), based on the INAVO-120 trial (AGO ++) [54].
–Alpelisib (not available in Germany) in combination with fulvestrant after prior endocrine therapy from second-line treatment onward, based on the SOLAR-1 trial (AGO ±) [55].

Accordingly, mutation status should be known no later than after first-line therapy; in endocrine-resistant patients, already at the time of diagnosis of distant disease (AGO ++).

Activation of the PI3K/AKT/PTEN pathway may also occur through AKT mutations or loss of the inhibitory tumor suppressor PTEN, observed in approximately 2–3% and 5–10% of patients with MBC, respectively [56]. Again, high concordance between primary and metastatic lesions has been demonstrated. Like PIK3CA, AKT alterations can be detected using ctDNA. However, due to the lower sensitivity of ctDNA-based assays for PTEN deletions, tumor tissue should preferably be used for PTEN assessment. Based on the CAPItello-291 trial, the only recently approved AKT inhibitor is capivasertib, which is used in combination with fulvestrant in patients with disease progression under endocrine therapy and alterations in the PI3K/AKT/PTEN pathway (AGO +) [57]. Therefore, PIK3CA, AKT, and PTEN status should be determined no later than at the start of second-line therapy (AGO +).

Under the selective pressure of endocrine therapy, mutations may arise in the ESR1 gene which encodes the estrogen receptor. These mutations typically affect the ligand-binding domain (especially D538G, Y537S, Y537N, Y537C, and E380) and result in ligand-independent receptor activation [58]. While ESR1 mutations are rare in untreated tumors, they are detected in approximately 20–40% of endocrine-pretreated MBC [59]. Therefore, to accurately capture current mutation status, testing should not be performed on archived tissue. Instead, liquid biopsy (ctDNA analysis) represents an optimal approach to assess current mutation status and to address tumor heterogeneity across metastatic sites to track resistance against aromatase inhibitors (AGO ++) [[60], [61], [62]]. As a previously negative result may no longer reflect the current tumor biology, ctDNA based ESR1 testing should be considered at each disease progression during endocrine-based treatment.

Oral selective estrogen receptor degraders (SERDs) can overcome endocrine resistance driven by ESR1 mutations. The oral SERDs elacestrant (EMERALD trial) and imlunestrant (EMBER-3 trial) are approved as monotherapy for the treatment of HR-positive/HER2-negative MBC with ESR1 mutations after resistance to prior endocrine therapy (AGO +) [63,64]. The benefit from elacestrant appears to be more pronounced in patients with longer prior exposure to CDK4/6 inhibitor therapy, particularly ≥12 months. The oral SERD giredestrant, has demonstrated increased efficacy when combined with everolimus also in ESR1 wild-type HR-positive/HER2-negative MBC [65].

In patients with metastatic breast cancer lacking standard therapeutic options, evaluation of additional molecular targets using extended NGS panels may be considered within the framework of a multidisciplinary molecular tumor board. The European Society of Medical Oncology (ESMO) Scale for Clinical Actionability of Molecular Targets (ESCAT) defines six levels of clinical evidence for molecular targets according to their implications for patient management [66]. Additional treatment options with ESCAT level I-II evidence are listed in Table 5 and include olaparib in patients with somatic BRCA mutations, checkpoint inhibitors in patients with microsatellite instability (MSI) or high tumor mutational burden (TMB) (AGO +), neurotrophic tyrosine receptor kinase (NTRK) inhibitors in patients with NTRK gene fusions (AGO +), and tyrosine kinase inhibitors in patients with HER2 mutations (AGO +/−) [12].

**Table 5:** Treatment relevant mutation diagnostic in metastatic breast cancer.

Altered genes	Therapeutic relevance	Gene region	Material	AGO
BRCA1, BRCA2	Olaparib, Talazoparib	All exons	Germline: Blood cells	++
	Olaparib		Somatic: Primary tumor, metastases	+
PALB2	Olaparib		Germline: Blood cells	+
PIK3CA	Alpelisib Inavolisib	Exons 7, 9, 20	Primary tumor, metastases, ctDNA	++
AKT1, PTEN, PIK3CA	Capivasertib		Primary tumor, metastases, ctDNA	+
ESR1	Resistance against AI	Exons 4, 7, 8	Metastases, ctDNA	+
	Elacestrant, Imlunestrant, Giredestrant			++
NTRK gene fusion	Larotrectinib, Entrectinib, Reprotectinib	Fusion- and splice variants	Primary tumor, metastases, particulary secretory BC	+
MSI	Pembrolizumab	Microsatellite-instability	Primary tumor, metastases	+
TMB	Checkpoint-Inhibitors	Tumor mutational burden	Primary tumor, metastases	+
HER2-mutations	Tyrosin Kinase Inhibitors	Kinase- and extracellular domains; S310, L755, V777, Y772_A775dup	Primary tumor, metastases	+/−

AGO, Arbeitsgemeinschaft Gynäkologische Onkologie; AI, aromatase inhibitor; BC, breast cancer; BRCA1/2, breast cancer1/2 gene; ctDNA, circulating tumor deoxyribonucleic acid; ESR1, estrogen receptor alpha gene; HER2, human epidermal growth factor receptor 2; HR, hormone receptor; NTRK, neurotrophic tyrosine receptor kinase; PALB2, partner and localizer of BRCA2; PIK3CA, phosphatidylinositol-4,5-bisphosphate 3-kinase catalytic subunit alpha, MSI, microsatellite-instability.

## 7 Circulating biomarkers

As part of tumor progression, individual tumor cells may enter the bloodstream. Moreover, tumor-specific molecules may be released into the circulation, particularly during tumor growth and disease progression. Circulating tumor DNA (ctDNA) consists of tumor-derived DNA fragments present in blood plasma. In principle, two main approaches to detect ctDNA detection are used: tumor-informed and tumor-uninformed (tumor-agnostic) assays [67]. Tumor-informed ctDNA assays are based on prior sequencing of the individual patient's tumor tissue to identify tumor-specific somatic mutations that are then tracked in the plasma. Tumor-informed approaches are mainly investigated in EBC for minimal residual disease (MRD) detection and early relapse monitoring (AGO +/−) [[68], [69], [70], [71]]. Currently, these assays should be offered only within clinical trials, such as the SURVIVE study, which investigates the use of ctDNA monitoring to tailor surveillance strategies [72].

Tumor-uninformed (tumor-agnostic) ctDNA assays do not require prior knowledge of the tumor's genomic profile and instead screen plasma for predefined panels of common alterations [73]. In breast cancer (BC), these assays are primarily used in the metastatic setting to identify actionable somatic alterations (e.g., in PIK3CA, ESR1, and AKT1) that guide treatment selection or to monitor the emergence of ESR1 mutations during endocrine therapy (AGO ++) [64,74,75]. The randomized phase III PADA-1 and SERENA-6 trials demonstrated that an early switch from an aromatase inhibitor to a selective estrogen receptor degrader (SERD), while maintaining CDK4/6 inhibition, delays disease progression in patients with HR-positive, HER2-negative breast cancer who develop ctDNA-detected ESR1 mutations before radiographic progression [61,75].

## 8 Conclusion

A thorough understanding of prognostic and predictive biomarkers and the appropriate timing of their assessment is essential for selecting an optimal treatment strategy. With an increasing number of targeted therapies, accurate prognostic evaluation and prediction of treatment response are increasingly important for identifying patients who derive maximal therapy response while avoiding unnecessary toxicity. To capture dynamic changes in tumor biology and biomarker status, longitudinal molecular assessment through re-biopsy of residual or recurrent disease and/or repeated liquid biopsy should be considered whenever feasible, particularly when changes in biomarker status are likely to influence treatment selection. Continued research into biomarkers and their integration into therapeutic decisions will further refine risk stratification and therapeutic personalization in the era of precision medicine.

## CRediT authorship contribution statement

**Andreas Hartkopf:** Conceptualization, Writing – original draft, Writing – review & editing. **Ute-Susann Albert:** Conceptualization, Writing – review & editing. **Rupert Bartsch:** Conceptualization, Writing – review & editing. **Ingo Bauerfeind:** Conceptualization, Writing – review & editing. **Maggie Banys-Paluchowski:** Conceptualization, Writing – review & editing. **Vesna Bjelic-Radisic:** Conceptualization, Writing – review & editing. **Jens-Uwe Blohmer:** Conceptualization, Writing – review & editing. **Peter Dall:** Conceptualization, Writing – review & editing. **Eva Maria Fallenberg:** Conceptualization, Writing – review & editing. **Peter A. Fasching:** Conceptualization, Writing – review & editing. **Michael Friedrich:** Conceptualization, Writing – review & editing. **Oleg Gluz:** Conceptualization, Writing – review & editing. **Nadia Harbeck:** Conceptualization, Writing – review & editing. **Jörg Heil:** Conceptualization, Writing – review & editing. **Juliane Hörner-Rieber:** Conceptualization, Writing – review & editing. **Jens Huober:** Conceptualization, Writing – review & editing. **Wolfgang Janni:** Conceptualization, Writing – review & editing. **Hans-Heinrich Kreipe:** Conceptualization, Writing – review & editing, Writing – review & editing, Conceptualization. **David Krug:** Conceptualization, Writing – review & editing. **Thorsten Kühn:** Conceptualization, Writing – review & editing. **Sherko Kümmel:** Conceptualization, Writing – review & editing. **Sibylle Loibl:** Conceptualization, Writing – review & editing. **Diana Lüftner:** Conceptualization, Writing – review & editing. **Michael Patrick Lux:** Conceptualization, Writing – review & editing. **Nicolai Maass:** Conceptualization, Writing – review & editing. **Marion Tina van Mackelenbergh:** Conceptualization, Writing – review & editing. **Christoph Mundhenke:** Conceptualization, Writing – review & editing. **Tjoung-Won Park-Simon:** Conceptualization, Writing – review & editing. **Mattea Reinisch:** Conceptualization, Writing – review & editing. **Toralf Reimer:** Conceptualization, Writing – review & editing. **Kerstin Rhiem:** Conceptualization, Writing – review & editing. **Achim Rody:** Conceptualization, Writing – review & editing. **Andreas Schneeweiss:** Conceptualization, Writing – review & editing. **Florian Schütz:** Conceptualization, Writing – review & editing. **Hans-Peter Sinn:** Conceptualization, Writing – review & editing. **Christine Solbach:** Conceptualization, Writing – review & editing. **Erich-Franz Solomayer:** Conceptualization, Writing – review & editing. **Elmar Stickeler:** Conceptualization, Writing – review & editing. **Marc Thill:** Conceptualization, Writing – review & editing. **Michael Untch:** Conceptualization, Writing – review & editing. **Isabell Witzel:** Conceptualization, Writing – review & editing. **Achim Wöckel:** Conceptualization, Writing – review & editing. **Rachel Wuerstlein:** Conceptualization, Writing – review & editing. **Nina Ditsch:** Conceptualization, Writing – review & editing. **Tanja Fehm:** Conceptualization, Writing – review & editing. **Volkmar Müller:** Conceptualization, Writing – review & editing. **Marcus Schmidt:** Conceptualization, Writing – original draft, Writing – review & editing.

## Declaration of competing interest

The authors declare the following financial interests/personal relationships which may be considered as potential competing interests: Andreas Hartkopf reports relationships with Amgen, Roche, Novartis, Lilly, MSD, AstraZeneca, Agendia, DaichiiSankyo, Seagen, GSK, ExactScience, Gilead, Menarini Stemline, Pfizer, Eisai, Veracyte, Onkowissen, Springer-Nature, Thieme, Pierre Fabre that include consultation or advisory fees and travel reimbursement from Roche, Novartis, Lilly, AstraZeneca, GSK, ExactScience, Gilead, Menarini Stemline, Pfizer, and DaichiiSankyo. He has received institutional research funding from ExactScience, Veracyte, the German Cancer Aid (DKG), the German Research Foundation (DFG), Claudia Schilling Foundation, Digitaloffensive BW and the Klaus Tschiara Foundation. Ute-Susann Albert recived honoraria for lectures and/or consulting from AstraZeneca, Novartis, Pfizer, ATP-Verlag, IQTIG and Bundesamt für Strahlenschutz. Rupert Bartsch has received honoraria from Amgen, Astra-Zeneca, BMS, Daiichi-Sankyo, Eisai, Eli-Lilly, Gilead, Gruenenthal, MSD, MedMedia, Novartis, Pfizer, Pierre-Fabre, Roche, and Stemline, research support from Astra-Zeneca, Daiichi-Sanyko, and MSD, and travel support from Astra-Zeneca, Daiichi-Sankyo, Eli-Lilly, Gilead, MSD, Novartis, Pfizer, and Roche. Ingo Bauerfeind reports lecture fees from Lilly and Stemline. Maggie Banys-Paluchowski reports relationships with Roche, Novartis, Pfizer, pfm, Eli Lilly, Onkowissen, Seagen, AstraZeneca, Eisai, Amgen, Samsung, Canon, MSD, GSK, Daiichi Sankyo, Gilead, Sirius Medical, Syantra, resitu, Pierre Fabre, ExactSciences, and Aurikamed for consultation or advisory fees; study support from Korean Breast Cancer Society, Eugen & Irmgard Hahn Stiftung, EndoMag, Mammotome, MeritMedical, Sirius Medical, Gilead, Hologic, ExactSciences, Claudia von Schilling Stiftung, Damp Stiftung, and Ehmann Stiftung Savognin and travel reimbursement from Eli Lilly, ExactSciences, Pierre Fabre, Pfizer, Daiichi Sankyo, Roche, and Stemline. Jens-Uwe Blohmer reports relationships with AstraZeneca, Daiichi Sankyo, Fresenius Kabi, Gilead, Lilly, MSD, Novartis, Pfizer, and Roche that include consultation or advisory fees. Peter Dall reports honoraria for lectures and advisory boards from Novartis, Pierre Fabré, MSD, Lilly, AstraZeneca, Gilead, Daiichi Sankyo, Eisai, and Polytech. Eva Maria Fallenberg reports a research grant from DFG and advisory, lecture fees and travel support from Bayer Healthcare, Guerbet, Siemens, BD, Roche, EUSOBI, ESOR, ESMO, ESO, B-Rayz, and RANZR. Peter A. Fasching reports participation on a Data Safety Monitoring Board or Advisory Board from Novartis, Pfizer, Roche, Daiichi-Sankyo, AstraZeneca, Lilly, Eisai, Merck Sharp & Dohme, Pierre Fabre, SeaGen, Agendia, Sanofi Aventis, Gilead, and Mylan; honoraria for lecture, speakers bureaus, manuscript writing or educational events from Novartis, Pfizer, Roche, Daiichi-Sankyo, AstraZeneca, Lilly, Eisai, Merck Sharp & Dohme, Pierre Fabre, SeaGen, Agendia, Sanofi Aventis, Gilead, and Mylan; consultation or advisory fees from Novartis, Pfizer, Roche, Daiichi-Sankyo, AstraZeneca, Lilly, Eisai, Merck Sharp & Dohme, Pierre Fabre, SeaGen, Agendia, Sanofi Aventis, Gilead, and Mylan; Medical Writing from Merck and institutional fees from Biontech, Cepheid, and Pfizer. Oleg Gluz recived honoraria for lectures and/or consulting from Amgen, AstraZeneca, Daiichi Sanyko, Eisai, Gilead Science, Lilly, MSD, Novartis, Pfizer, Roche, Seagen, Exact Sciences, Agendia, MedConcept, and GynUpdate and is minority share holder of Westdeutsche Studiengruppe (WSG). Nadia Harbeck recived honoraria for lectures and/or consulting from AstraZeneca, Daiichi-Sankyo, Gilead, High5Oncology, IQVIA, Lilly, MEDSCAPE, Menarini-Stemline, MSD, Novartis, Pierre-Fabre, Pfizer, Roche, Springer, and Viatris and is Co-Director West German Study Group (WSG). Juliane Hörner-Rieber reports speaker fees from ViewRay Inc., Pfizer Inc., Accuray Sarl, Sanofi, and Astra Zeneca, travel support from Varian Medical Systems, and research grants from IntraOP Medical and Varian Medical Systems. Wolfgang Janni recived research grants and/or honoraria from AstraZeneca, Cellgene, Chugai, DaiichiSankyo, Eisai, ExactScience, Gilead, GSK, Guardant Health, Janssen, Lilly, Menarini Stemline, MSD, NeoGenomics, Novartis, Pfizer, Roche, Sanofi-Aventis, and Seagen. Hans-Heinrich Kreiper reports a relationship with Lilly that include consultation or advisory fees and lecture fees from AstraZeneca, Roche, Daiichi Sankyo, and Pfizer. David Krug received honoraria from Astra Zeneca, best practice onkologie, ESO, ESMO, Gilead, med update, Merck Sharp & Dohme, Novartis, onkowissen, Pfizer, and PINK gegen Brustkrebs, consultation or advisory fees from Gilead, and has received institutional research funding from Stiftung Deutsche Krebshilfe and Merck KGaA. Thorsten Kühn recived honoraria from MSD, Exact Sciences, Novartis, Lilly, Merit Medical, Sirius Medical, and Endomag. Sherko Kuemmel recived consulting or advisory fees from Roche/Genentech, Novartis, AstraZeneca, Amgen, Daiichi Sankyo, Pfizer, MSD Oncology, Lilly, Sonoscape, Gilead Sciences, Seagen, Agendia, Stryker, Hologic, PINK, Exact Sciences, Stemline; speaking and lecture fees from Novartis and travel support from Roche, Daiichi Sankyo, and Gilead Sciences and uncompensated relationships with West-Deutsche-Studiengruppe (WSG). Sibylle Loibl reorts grants and/or honorarium for adboards and/or contracts from the following entities/all paid to institution: AZ,Abbvie, Agendia, Amgen, BionTech, Celgene/BMS, Cellcuity, DSI, Exact Science, Gilead, GSK, Incyte, Lilly, Medscape, Molecular Health, MSD, Novartis, Pierre Fabre, Pfizer, Relay, Roche, Sanofi, Seagen, Stemline/Menarini, Olema, Bayer, Bicycle, JAZZ Pharmaceuticals, BeiGene, Jiangsu Hengrui, MICE ideas, Corcept Therapeutics, Peerview, AICME, and Chugai Pharmaceuticals; travel reimbursement from DSI, Gilead, Roche, Lilly, ESMO, SGBCC, ASCO, AGO Kommission Mamma; the following patents planned, issued or pending: EP14153692.0/EP21152186.9/EP18209672/EP24210258; and roylties from VM Scope. Diana Lueftner reports relationsships with Abbvie, Amgen, AstraZeneca, BMS, Daiichi Sankyo, Eli Lilly, Exact Sciences, Genmab, Gilead, GSK, high5md, Loreal, MSD, Novartis, onkowissen.de, Pfizer, Roche, Seagen, and TEVA that include adivisory and lecture fees. MIchael Patrick Lux reports relationships with Abbvie, ClinSol GmbH & Co. KG, Lilly, Pfizer, Roche, MSD, Hexal, Novartis, AstraZeneca, Eisai, Exact Sciences, Agendia, Daiichi Sankyo, Grünenthal, Gilead, Stemline, Samantree and Endomag for that include consultation or advisory fees, lecture fees and travel reimbursement. Nicolai Maass reports relationships with Amgen, AstraZeneca, Clovis, Daiichi Sankyo, GSK, Lilly, MSD, Novartis, Pierre Fabre, Pfizer, Roche, Seagen Lecture: Astra Zeneca, Daiichi Sankyo, GSK, Lilly, Novartis, Pfizer, and Roche for consultation or advisory fees. Marion Thina van Mackelenbergh reports honoraria from Amgen, AstraZeneca, Daiichi Sankyo, Exact Sciences, Gilead, GSK, Jenapharm, Lilly, Menarini Stemline, Molecular Health, Mylan, MSD, Novartis, Pfizer, PierreFabre, Roche, and Seagen and travel grants from AstraZeneca, Daiichi Sankyo, Gilead, Lilly, Novartis, and Roche. Christoph Mundhenke reports relationsships with AstraZeneca, Pfizer, Daiichi Sankyo, Seagen, and Novartis that include consultation or advisory fees and speaking and lecture fees from Pfize and Novartis. Tjoung-Won Park-Simon recived honoraria for lectures and/or consulting from Roche, AstraZeneca, GSK, Pfizer, Lilly, MSD, Exact Sciences, Daiichi Sankyo, Seagen, Novartis, Gilead Science, NCO, Onkowissen, Exact Sciences, and Seagen; and travel reimbursement from Roche, AstraZeneca, Pfizer, Lilly, Daiichi Sankyo, Gilead, and PierreFabre. Mattea Reinisch received consultation or advisory fees from AstraZeneca, Daiichi Sankyo, Gilead, jenaPharma, Lilly, MSD, Novartis, Pfizer, Roche, OnkowissenTV, Sidekickhealth, Seagen, Streamed Up, Somatex, Wiley, and Walters Kluwer and travel support from AstraZeneca, Daiichi Sankyo, Gilead, Lilly, Novartis, Pfizer, and Roche. Toralf Reimer reports trial funding from German Cancer Aid and Else Kroener-Fresenius-Stiftung and advisory fees from MSD, Novartis, Myriad Lecture: Pfizer, Novartis, Roche, and AstraZeneca. Kerstin Riehm received honoraria from Pfizer, served on advisory boards for AstraZeneca and Jenapharm and has received institutional research funding from the Federal Ministry of Research, Technology and Space. Achim Rody reports relationships with AstraZeneca, Novartis, Roche, Exact Sciences, Pierre Fabre, Lilly, Seagen, Amgen, MSD, and Gilead that include consulting or advisory fees, speaking and lecture fees from Pfizer and Celgene, and trial funding from Eisai. Andreas Schneeweiss recived honoraria from Amgen, AstraZeneca, Aurikamed, Bayer, Celgene, ClinSol, Clovis Oncology, coma, UroGyn, Connectmedica, Daiichi Sankyo, Gilead, GSK, if-kongress, I-MED, iOMEDICO, Lilly, MCI Deutschland, med publico, Menarini Stemline, Metaplan, MSD, Mylan, NeoConnect, NanoString Technologies, Novartis, onkowissen.de, Pfizer, Pierre Fabre, promedicis, Roche, Seagen, streamedup, SYNLAB, Tesaro and tavel reimbursementfrom AstraZeneca, Celgene, Daiichi Sankyo, Gilead, Pfizer, and Roche. Florian Schuetz reports lecture fees from Amgen, AstraZeneca, Daiichi Sankyo, Eisai, ExactSciences, Gilead, Lilly, MSD, Novartis, ClinSol, Pfizer, and Roche Pharma, advisory fees from Lilly, MSD, Gilead, Atheneum Partners, ClinSol and travel reimursement from Lilly and Gilead. Hans-Peter Sinn reports relationships with Astra Zeneca, Exact Sciences, Daiichi Sankyo Lecture: AstraZeneca, and Diacentus that include consultation or advisory fees and trial funding from AstraZeneca. Christine Solbach recived speaking and lecture fees from Dialog Service GmbH, Jörg Eickeler, MedConcept, Medela, Samsung, Novartis, Roche, Pfizer, and LÄKH. Elmar Stickeler reports relationsships with Amgen, Astra Zeneca, Gilead, Iomedico, Lilly, MSD,Novartis, Seagen, and Roche that include consultation or advisory fees and speaking and lecture fees from Astra Zeneca, BSH Düsseldorf, Gilead, Iomedico, MSD, Novartis, Onkowissen, Pfizer, PharmaMar, and Roche. Marc Thill reports relationships with Agendia, Amgen, AstraZeneca, Aurikamed, Becton/Dickinson, ClearCut, Daiichi Sankyo, Eisai, Exact Sciences, Gilead Science, GSK, Johnson and Johnson, Lilly, MSD, Neodynamics, Novartis, Onkowissen, Onqo Health, Organon, Pfizer, pfm Medical, Pierre-Fabre, Roche, RTI Surgical, Saman Tree, Seagen, Sirius Medical, and Sysmex for consultation or advisory fees; manuscript support from Amgen, Cairn Surgical ClearCut, Lilly, Organon, pfm medical, Roche, and Servier; travel reimbursement from Amgen, Art Tempi, AstraZeneca, Clearcut, Clovis, Daiichi Sankyo, Eisai, Exact Sciences, Gilead, Hexal, I-Med-Institute, Lilly, Menarini Stemline, MSD, Neodynamics, Novartis, Pfizer, pfm Medical, Roche, RTI Surgical, Seagen, Zuellig Pharma, Pierre Fabre, and Sirius Medical; speaking and lecture fees from Agendia, Amgen, Art Tempi, AstraZeneca, Eisai, Endomag, Exact Sciences, Gilead Science, GSK, Hexal, I-Med-Institute, Jörg Eickeler, Laborarztpraxis Walther et al., Lilly, Medscape, Menarini Stemline, MSD, Novartis, Onkowissen, Pfizer, pfm medical, Roche, Seagen, Sirius Medical, StreamedUp, Sysmex, Vifor, Viatris, and Zuellig Pharma; trial funding from Endomag and Exact Sciences; and institutional trial funding from AstraZeneca, Biom’Up, CairnSurgical, Clearcut, Neodynamics, Novartis, Novus Scientific, pfm medical, Roche, and RTI Surgical. Michael Untch recived institutional fees for lectures and presentations from Astra Zeneca, Daiichi Sankyo, Lilly, EurBio Scientific, Myriad Genetics, Novartis; Pfizer, Roche, Sanofi Aventis, Menarini Stemline, and MSD Merck and institutional honoraria for advisory boards from Astra Zeneca, Daiichi Sankyo, Lilly, MSD Merck, Myriad Genetics, Novartis, Pierre Fabre, Pfizer, Gilead, Roche; Sanofi Aventis, Seagen, Menarini Stemline, and BeOne Germany. Isabell Witzel reports relatioships with Astra Zeneca, Lilly, Seagen, Daiichi Sankyo, Gilead, Pfizer, Novartis, and Onkowissen and travel reimbursement from Roche and Lilly. Amgen, AstraZeneca, Aurikamed, Celgene, Eisai, Lilly, Novartis, Pfizer, Roche, Tesaro, Sirtex, MSD, Exact Sciences, Pierre Fabre, Clovis, Organon, Daiji Sankyo, Seagen, Stemline, Gilead. Rachel Wuerstlein reports relationships with Agendia, Amgen, APOGHEVA, Aristo, Astra Zeneca, Bayer, Clinsol, Daiichi-Sankyo, Eisai, Esteve, Exact Sciences, Gilead, Hexal/Sandoz, Jenapharm, Lilly, Lukon, MSD, Mundipharma, Mylan, Nanostring, Novartis, Onkowissen, Paxman, Palleos, Pfizer, Pierre Fabre, PINK, Roche, Seagen, Sidekick, Stemline, Tesaro Bio, Teva, Veracyte, Viatris, Wiley, and Thieme for consultation, advisory fees or lectures. Nina Ditsch reports relationships with Gilead, Lilly, MSD, Novartis, Pfizer, Roche, Exact Sciences, Merit-Medical, and Pierre-Fabre for consultation or advisory fees, speaking and lecture fees from AstraZeneca, Fa. Aurikamed, COCS GmbH, Daiichi-Sankyo, ESMO International, Novartis, Roche, Lilly, Pfizer, Gilead, Novartis, Onkowissen, Jörg Eickeler Kongress, if-Kongress, Fa. Titanium Textiles, RG Kongress, Onkowissen, ClinSol GmbH, Kelcon GmbH, Dt. Apotheker Verlag, Fa. Menarini-Stemline, FA. KUKM Kongress, Fa. BMMC Kongress, Fa. Gapinsaat, VHS Augsburg, Fa. Medi-Seminar GmbH, RRC-Kongress, and I-Med. Institute Berlin, manuscript writing from pfm Medical ag and trial funding from Gilead, BZKF, and pfm Medical ag. Tanja Fehm recived consultation or advisory fees from Medconcept, Onkowissen, and FOMF and travel supports from Pfizer, Roche, and Daichii Sankyo. Volkmar Mueller recived speaker honoraria from Astra Zeneca, arsTempi, Daiichi-Sankyo, Eisai, Pfizer, MSD, Medac, Novartis, Roche, Seagen, Onkowissen, high5 Oncology, Lilly, Medscape, Gilead, Pierre Fabre, and iMED Institute; consultancy honoraria from Roche, Pierre Fabre, PINK, ClinSol, Novartis, MSD, Daiichi-Sankyo, Eisai, Lilly, Seagen, Gilead, and Stemline; institutional research support from Novartis, Roche, Seagen, Genentech, Astra Zeneca, Pfizer; and travel reimbursement from Astra Zeneca, Roche, Pfizer, Daiichi Sankyo, and Gilead. Marcus Schmidt recived personal fees from AstraZeneca, BioNTech, Daiichi Sankyo, Eisai, Eurobio, Exact Sciences, Gilead, Lilly, Menarini-Stemline, Molecular Health, MSD, Novartis, Pantarhei Bioscience, Pfizer, Pierre Fabre, and Roche. His institution has received research funding from AstraZeneca, BioNTech, Eisai, Genentech, the German Breast Group, Novartis, Palleos, Pantarhei Bioscience, Pfizer, Pierre Fabre, and Roche. Andreas Hartkopf reports a patent pending to 5402P70. Marcus Schmidt reports patents issued to EP 2390370 B1, EP 2951317 B1. Sibylle Loibl reports patents planned, issued or pending to EP14153692.0/EP21152186.9/EP18209672 and roylties from VM Scope. Given their role as Specialty Editors of *The Breast*, Jörg Heil and Volkmar Müller had no involvement in the peer-review of this article and has no access to information regarding its peer-review. Full responsibility for the editorial process for this article was delegated to another journal editor.

## References

[1] Brandt J., Garne J.P., Tengrup I., Manjer J. (2015). Age at diagnosis in relation to survival following breast cancer: a cohort study. World J Surg Oncol.

[2] Chan D.S.M., Vieira A.R., Aune D., Bandera E.V., Greenwood D.C., McTiernan A. (2014). Body mass index and survival in women with breast cancer-systematic literature review and meta-analysis of 82 follow-up studies. Ann Oncol.

[3] Pan H., Gray R., Braybrooke J., Davies C., Taylor C., McGale P. (2017). 20-Year risks of breast-cancer recurrence after stopping endocrine therapy at 5 years. N Engl J Med.

[4] Moran M.S., Schnitt S.J., Giuliano A.E., Harris J.R., Khan S.A., Horton J. (2014). Society of surgical oncology-American society for radiation oncology consensus guideline on margins for breast-conserving surgery with whole-breast irradiation in stages I and II invasive breast cancer. Ann Surg Oncol.

[5] Cortazar P., Zhang L., Untch M., Mehta K., Costantino J.P., Wolmark N. (2014). Pathological complete response and long-term clinical benefit in breast cancer: the CTNeoBC pooled analysis. Lancet.

[6] Loibl S., Weber K., Huober J., Krappmann K., Marme F., Schem C. (2018). Risk assessment after neoadjuvant chemotherapy in luminal breast cancer using a clinicomolecular predictor. Clin Cancer Res.

[7] Yau C., Osdoit M., van der Noordaa M., Shad S., Wei J., de Croze D. (2022). Residual cancer burden after neoadjuvant chemotherapy and long-term survival outcomes in breast cancer: a multicentre pooled analysis of 5161 patients. Lancet Oncol.

[8] Rakha E.A., Aleskandarani M., Toss M.S., Green A.R., Ball G., Ellis I.O. (2018). Breast cancer histologic grading using digital microscopy: concordance and outcome association. J Clin Pathol.

[9] Zhong Y.M., Tong F., Shen J. (2022). Lympho-vascular invasion impacts the prognosis in breast-conserving surgery: a systematic review and meta-analysis. BMC Cancer.

[10] Gray E., Marti J., Brewster D.H., Wyatt J.C., Hall P.S., Group S.A. (2018). Independent validation of the PREDICT breast cancer prognosis prediction tool in 45,789 patients using Scottish cancer registry data. Br J Cancer.

[11] Richman J., Ring A., Dowsett M., Sestak I. (2021). Clinical validity of clinical treatment score 5 (CTS5) for estimating risk of late recurrence in unselected, non-trial patients with early oestrogen receptor-positive breast cancer. Breast Cancer Res Treat.

[12] Gennari A., Andre F., Barrios C.H., Cortes J., de Azambuja E., DeMichele A. (2021). ESMO clinical practice guideline for the diagnosis, staging and treatment of patients with metastatic breast cancer. Ann Oncol.

[13] Curigliano G., Burstein H.J., Gnant M., Loibl S., Cameron D., Regan M.M. (2023). Understanding breast cancer complexity to improve patient outcomes: the St Gallen international consensus conference for the primary therapy of individuals with early breast cancer 2023. Ann Oncol.

[14] Schrijver W., Suijkerbuijk K.P.M., van Gils C.H., van der Wall E., Moelans C.B., van Diest P.J. (2018). Receptor conversion in distant breast cancer metastases: a systematic review and meta-analysis. J Natl Cancer Inst.

[15] Walter V., Fischer C., Deutsch T.M., Ersing C., Nees J., Schutz F. (2020). Estrogen, progesterone, and human epidermal growth factor receptor 2 discordance between primary and metastatic breast cancer. Breast Cancer Res Treat.

[16] Hennigs A., Riedel F., Gondos A., Sinn P., Schirmacher P., Marme F. (2016). Prognosis of breast cancer molecular subtypes in routine clinical care: a large prospective cohort study. BMC Cancer.

[17] Gaudet M.M., Gierach G.L., Carter B.D., Luo J., Milne R.L., Weiderpass E. (2018). Pooled analysis of nine cohorts reveals breast cancer risk factors by tumor molecular subtype. Cancer Res.

[18] Davies C., Godwin J., Gray R., Clarke M., Cutter D. (2011). Relevance of breast cancer hormone receptors and other factors to the efficacy of adjuvant tamoxifen: patient-level meta-analysis of randomised trials. Lancet.

[19] Campbell F.C., Blamey R.W., Elston C.W., Morris A.H., Nicholson R.I., Griffiths K. (1981). Quantitative oestradiol receptor values in primary breast cancer and response of metastases to endocrine therapy. Lancet.

[20] van Mackelenbergh M.T., Denkert C., Nekljudova V., Karn T., Schem C., Marme F. (2018). Outcome after neoadjuvant chemotherapy in estrogen receptor-positive and progesterone receptor-negative breast cancer patients: a pooled analysis of individual patient data from ten prospectively randomized controlled neoadjuvant trials. Breast Cancer Res Treat.

[21] Allison K.H., Hammond M.E.H., Dowsett M., McKernin S.E., Carey L.A., Fitzgibbons P.L. (2020). Estrogen and progesterone receptor testing in breast cancer: ASCO/CAP guideline update. J Clin Oncol.

[22] Wolff A.C., Hammond M.E.H., Allison K.H., Harvey B.E., Mangu P.B., Bartlett J.M.S. (2018). Human epidermal growth factor receptor 2 testing in breast cancer: American society of clinical oncology/college of American pathologists clinical practice guideline focused update. J Clin Oncol.

[23] von Minckwitz G., Untch M., Blohmer J.U., Costa S.D., Eidtmann H., Fasching P.A. (2012). Definition and impact of pathologic complete response on prognosis after neoadjuvant chemotherapy in various intrinsic breast cancer subtypes. J Clin Oncol.

[24] Wolff A.C., Somerfield M.R., Dowsett M., Hammond M.E.H., Hayes D.F., McShane L.M. (2023). Human epidermal growth factor receptor 2 testing in breast cancer: ASCO-college of American pathologists guideline update. J Clin Oncol.

[25] Modi S., Jacot W., Yamashita T., Sohn J., Vidal M., Tokunaga E. (2022). Trastuzumab deruxtecan in previously treated HER2-Low advanced breast cancer. N Engl J Med.

[26] Bardia A., Hu X., Dent R., Yonemori K., Barrios C.H., O'Shaughnessy J.A. (2024). Trastuzumab deruxtecan after endocrine therapy in metastatic breast cancer. N Engl J Med.

[27] Toli M.A., Liu X., Massa D., Lando S., Boman C., Tsiknakis N. (2025). Prognostic significance of tumour Ki-67 dynamics during neoadjuvant treatment in patients with breast cancer: a population-based cohort study. Lancet Reg Health Eur.

[28] Dowsett M., Nielsen T.O., A'Hern R., Bartlett J., Coombes R.C., Cuzick J. (2011). Assessment of Ki67 in breast cancer: recommendations from the international Ki67 in breast cancer working group. J Natl Cancer Inst.

[29] Smith I., Robertson J., Kilburn L., Wilcox M., Evans A., Holcombe C. (2020). Long-term outcome and prognostic value of Ki67 after perioperative endocrine therapy in postmenopausal women with hormone-sensitive early breast cancer (POETIC): an open-label, multicentre, parallel-group, randomised, phase 3 trial. Lancet Oncol.

[30] Nitz U.A., Gluz O., Kummel S., Christgen M., Braun M., Aktas B. (2022). Endocrine therapy response and 21-Gene expression assay for therapy guidance in HR+/HER2- early breast cancer. J Clin Oncol.

[31] Hartkopf A.D., Taran F.A., Wallwiener M., Walter C.B., Kramer B., Grischke E.M. (2016). PD-1 and PD-L1 immune checkpoint blockade to treat breast cancer. Breast Care.

[32] Cortes J., Rugo H.S., Cescon D.W., Im S.A., Yusof M.M., Gallardo C. (2022). Pembrolizumab plus chemotherapy in advanced triple-negative breast cancer. N Engl J Med.

[33] Schmid P., Adams S., Rugo H.S., Schneeweiss A., Barrios C.H., Iwata H. (2018). Atezolizumab and nab-paclitaxel in advanced triple-negative breast cancer. N Engl J Med.

[34] Rugo H.S., Loi S., Adams S., Schmid P., Schneeweiss A., Barrios C.H. (2021). PD-L1 immunohistochemistry assay comparison in atezolizumab plus nab-paclitaxel-treated advanced triple-negative breast cancer. J Natl Cancer Inst.

[35] Schmid P., Cortes J., Pusztai L., McArthur H., Kummel S., Bergh J. (2020). Pembrolizumab for early triple-negative breast cancer. N Engl J Med.

[36] Gnant M., Filipits M., Greil R., Stoeger H., Rudas M., Bago-Horvath Z. (2014). Predicting distant recurrence in receptor-positive breast cancer patients with limited clinicopathological risk: using the PAM50 risk of recurrence score in 1478 postmenopausal patients of the ABCSG-8 trial treated with adjuvant endocrine therapy alone. Ann Oncol.

[37] Gnant M., Sestak I., Filipits M., Dowsett M., Balic M., Lopez-Knowles E. (2015). Identifying clinically relevant prognostic subgroups of postmenopausal women with node-positive hormone receptor-positive early-stage breast cancer treated with endocrine therapy: a combined analysis of ABCSG-8 and ATAC using the PAM50 risk of recurrence score and intrinsic subtype. Ann Oncol.

[38] Sparano J.A., Gray R.J., Makower D.F., Pritchard K.I., Albain K.S., Hayes D.F. (2018). Adjuvant chemotherapy guided by a 21-Gene expression assay in breast cancer. N Engl J Med.

[39] Kalinsky K., Barlow W.E., Gralow J.R., Meric-Bernstam F., Albain K.S., Hayes D.F. (2021). 21-Gene assay to inform chemotherapy benefit in node-positive breast cancer. N Engl J Med.

[40] Sestak I., Martin M., Dubsky P., Kronenwett R., Rojo F., Cuzick J. (2019). Prediction of chemotherapy benefit by EndoPredict in patients with breast cancer who received adjuvant endocrine therapy plus chemotherapy or endocrine therapy alone. Breast Cancer Res Treat.

[41] Cardoso F., van't Veer L.J., Bogaerts J., Slaets L., Viale G., Delaloge S. (2016). 70-Gene signature as an aid to treatment decisions in early-stage breast cancer. N Engl J Med.

[42] Stein R.C., Makris A., Macpherson I.R., Hughes-Davies L., Marshall A., Pinder S.E. (2026). First results from the OPTIMA phase III randomized non-inferiority trial of test-directed chemotherapy in patients with high clinical risk ER-positive HER2-negative early breast cancer. J Clin Oncol.

[43] Slamon D., Lipatov O., Nowecki Z., McAndrew N., Kukielka-Budny B., Stroyakovskiy D. (2024). Ribociclib plus endocrine therapy in early breast cancer. N Engl J Med.

[44] Fasching P.A., Loibl S., Hu C., Hart S.N., Shimelis H., Moore R. (2018). BRCA1/2 mutations and bevacizumab in the neoadjuvant treatment of breast cancer: response and prognosis results in patients with triple-negative breast cancer from the GeparQuinto study. J Clin Oncol.

[45] Tutt A.N.J., Garber J.E., Kaufman B., Viale G., Fumagalli D., Rastogi P. (2021). Adjuvant olaparib for patients with BRCA1- or BRCA2-Mutated breast cancer. N Engl J Med.

[46] Fasching P.A., Yadav S., Hu C., Wunderle M., Haberle L., Hart S.N. (2021). Mutations in BRCA1/2 and other panel genes in patients with metastatic breast cancer -Association with patient and disease characteristics and effect on prognosis. J Clin Oncol.

[47] Robson M., Im S.A., Senkus E., Xu B., Domchek S.M., Masuda N. (2017). Olaparib for metastatic breast cancer in patients with a germline BRCA mutation. N Engl J Med.

[48] Litton J.K., Rugo H.S., Ettl J., Hurvitz S.A., Goncalves A., Lee K.H. (2018). Talazoparib in patients with advanced breast cancer and a germline BRCA mutation. N Engl J Med.

[49] Tung N.M., Robson M.E., Li T., Nanda R., Shah P.D., Khoury K. (2026). TBCRC 048 (Olaparib expanded) expansion cohorts: phase II study of olaparib monotherapy for patients with metastatic breast cancer with germline mutations in PALB2 or somatic mutations in BRCA1 or BRCA2. J Clin Oncol.

[50] Hartkopf A.D., Grischke E.M., Brucker S.Y. (2020). Endocrine-resistant breast cancer: mechanisms and treatment. Breast Care.

[51] Browne I.M., Andre F., Chandarlapaty S., Carey L.A., Turner N.C. (2024). Optimal targeting of PI3K-AKT and mTOR in advanced oestrogen receptor-positive breast cancer. Lancet Oncol.

[52] Park L., Thompson S.L., Roose J., Lu Y., Chaki M., Lam C. (2024). Testing patterns and prevalence of PIK3CA, AKT1, and PTEN alterations among patients (pts) with HR+/HER2- metastatic breast cancer (mBC) in the US. J Clin Oncol.

[53] Anderson E.J., Mollon L.E., Dean J.L., Warholak T.L., Aizer A., Platt E.A. (2020). A systematic review of the prevalence and diagnostic workup of PIK3CA mutations in HR+/HER2- metastatic breast cancer. Int J Breast Cancer.

[54] Turner N.C., Im S.A., Saura C., Juric D., Loibl S., Kalinsky K. (2024). Inavolisib-based therapy in PIK3CA-Mutated advanced breast cancer. N Engl J Med.

[55] André F., Ciruelos E., Rubovszky G., Campone M., Loibl S., Rugo H.S. (2019). Alpelisib for PIK3CA-Mutated, hormone receptor–positive advanced breast cancer. N Engl J Med.

[56] Pereira B., Chin S.F., Rueda O.M., Vollan H.K., Provenzano E., Bardwell H.A. (2016). The somatic mutation profiles of 2,433 breast cancers refines their genomic and transcriptomic landscapes. Nat Commun.

[57] Turner N.C., Oliveira M., Howell S.J., Dalenc F., Cortes J., Gomez Moreno H.L. (2023). Capivasertib in hormone receptor-positive advanced breast cancer. N Engl J Med.

[58] Carausu M., Bidard F.C., Callens C., Melaabi S., Jeannot E., Pierga J.Y. (2019). ESR1 mutations: a new biomarker in breast cancer. Expert Rev Mol Diagn.

[59] Jeselsohn R., Yelensky R., Buchwalter G., Frampton G., Meric-Bernstam F., Gonzalez-Angulo A.M. (2014). Emergence of constitutively active estrogen receptor-alpha mutations in pretreated advanced estrogen receptor-positive breast cancer. Clin Cancer Res.

[60] Turner N.C., Swift C., Kilburn L., Fribbens C., Beaney M., Garcia-Murillas I. (2020). ESR1 mutations and overall survival on fulvestrant versus exemestane in advanced hormone receptor-positive breast cancer: a combined analysis of the phase III SoFEA and EFECT trials. Clin Cancer Res.

[61] Bidard F.C., Hardy-Bessard A.C., Dalenc F., Bachelot T., Pierga J.Y., de la Motte Rouge T. (2022). Switch to fulvestrant and palbociclib versus no switch in advanced breast cancer with rising ESR1 mutation during aromatase inhibitor and palbociclib therapy (PADA-1): a randomised, open-label, multicentre, phase 3 trial. Lancet Oncol.

[62] Fribbens C., Garcia Murillas I., Beaney M., Hrebien S., O'Leary B., Kilburn L. (2018). Tracking evolution of aromatase inhibitor resistance with circulating tumour DNA analysis in metastatic breast cancer. Ann Oncol.

[63] Jhaveri K.L., Neven P., Casalnuovo M.L., Kim S.B., Tokunaga E., Aftimos P. (2025). Imlunestrant with or without abemaciclib in advanced breast cancer. N Engl J Med.

[64] Bidard F.C., Kaklamani V.G., Neven P., Streich G., Montero A.J., Forget F. (2022). Elacestrant (oral selective estrogen receptor degrader) versus standard endocrine therapy for estrogen receptor-positive, human epidermal growth factor receptor 2-Negative advanced breast cancer: results from the randomized phase III EMERALD trial. J Clin Oncol.

[65] Mayer E., Tolaney S.M., Martin M., Vidal G.A., Moscetti L., Jhaveri K.L. (2025). Giredestrant (GIRE), an oral selective oestrogen receptor (ER) antagonist and degrader, + everolimus (E) in patients (pts) with ER-positive, HER2-negative advanced breast cancer (ER+, HER2– aBC) previously treated with a CDK4/6 inhibitor (i): primary results of the phase III evERA BC trial. Ann Oncol.

[66] Mateo J., Chakravarty D., Dienstmann R., Jezdic S., Gonzalez-Perez A., Lopez-Bigas N. (2018). A framework to rank genomic alterations as targets for cancer precision medicine: the ESMO scale for clinical actionability of molecular targets (ESCAT). Ann Oncol.

[67] Banys-Paluchowski M., Fehm T.N., Grimm-Glang D., Rody A., Krawczyk N. (2022). Liquid biopsy in metastatic breast cancer: current role of circulating tumor cells and circulating tumor DNA. Oncol Res Treat.

[68] Janni W., Rack B., Friedl T.W.P., Hartkopf A.D., Wiesmuller L., Pfister K. (2025). Detection of minimal residual disease and prediction of recurrence in breast cancer using a plasma-only circulating tumor DNA assay. ESMO Open.

[69] Loi S., Johnston S.R.D., Arteaga C.L., Graff S.L., Chandarlapaty S., Goetz M.P. (2024). Prognostic utility of ctDNA detection in the monarchE trial of adjuvant abemaciclib plus endocrine therapy (ET) in HR+, HER2-, node-positive, high-risk early breast cancer (EBC). J Clin Oncol.

[70] O'Leary B., Cutts R.J., Huang X., Hrebien S., Liu Y., Andre F. (2021). Circulating tumor DNA markers for early progression on fulvestrant with or without palbociclib in ER+ advanced breast cancer. J Natl Cancer Inst.

[71] Kuemmel S., Graeser M., Schmid P., Reinisch M., Feuerhake F., Volk V. (2025). Chemotherapy-free neoadjuvant pembrolizumab combined with trastuzumab and pertuzumab in HER2-enriched early breast cancer (WSG-KEYRICHED-1): a single-arm, phase 2 trial. Lancet Oncol.

[72] Braun T., Huesmann S., Pfister K., Friedl T.W.P., Hartkopf A., Pantel K. (2025). The SURVIVE study (NCT05658172): bringing breast cancer aftercare to the 21stcentury: study protocol of a Phase III clinical trial comparing liquid biopsy guided vs. Standard of care surveillance for intermediate to high-risk breast cancer survivors. PLoS One.

[73] Turner N.C., Kingston B., Kilburn L.S., Kernaghan S., Wardley A.M., Macpherson I.R. (2020). Circulating tumour DNA analysis to direct therapy in advanced breast cancer (plasmaMATCH): a multicentre, multicohort, phase 2a, platform trial. Lancet Oncol.

[74] Andre F., Ciruelos E.M., Juric D., Loibl S., Campone M., Mayer I.A. (2021). Alpelisib plus fulvestrant for PIK3CA-mutated, hormone receptor-positive, human epidermal growth factor receptor-2-negative advanced breast cancer: final overall survival results from SOLAR-1. Ann Oncol.

[75] Bidard F.C., Mayer E.L., Park Y.H., Janni W., Ma C., Cristofanilli M. (2025). First-line camizestrant for emerging ESR1-Mutated advanced breast cancer. N Engl J Med.

